# Development of a time-resolved fluorescence microsphere Eu lateral flow test strip based on a molecularly imprinted electrospun nanofiber membrane for determination of fenvalerate in vegetables

**DOI:** 10.3389/fnut.2022.957745

**Published:** 2022-09-20

**Authors:** Le Zhang, Yiliu Zheng, Hua Shao, Ming Xiao, Jianchun Sun, Maojun Jin, Fen Jin, Jing Wang, A. M. Abd El-Aty, Yongxin She

**Affiliations:** ^1^Institute of Quality Standards and Testing Technology for Agro-Products, Chinese Academy of Agricultural Sciences, Beijing, China; ^2^Academy of Agriculture and Forestry Sciences, Qinghai University, Xining, China; ^3^Inspection and Testing Center of Agricultural Products of Tibetan Autonomous Region, Lhasa, China; ^4^State Key Laboratory of Biobased Material and Green Papermaking, Qilu University of Technology, Shandong Academy of Sciences, Jinan, China; ^5^Department of Pharmacology, Faculty of Veterinary Medicine, Cairo University, Giza, Egypt; ^6^Department of Medical Pharmacology, Medical Faculty, Ataturk University, Erzurum, Turkey

**Keywords:** fenvalerate, molecularly imprinted polymer, electrospinning membrane, test strip, fluorescence

## Abstract

Fenvalerate residues in fruits and vegetables may result in biological immune system disorders. Current sensor detection methods are harsh due to the shortcomings of antibody preparation and preservation conditions. Therefore, developing a recognition material with strong specificity, good stability, and low cost is of practical significance in designing a sensitive, simple, and rapid method. This study used precipitation polymerization to synthesize molecularly imprinted polymers (MIPs). The MIP was prepared into a fiber membrane using the electrostatic spinning method. After that, the fenvalerate hapten-mouse IgG-Eu fluorescent probe was synthesized, and the side flow chromatography strip was constructed to determine fenvalerate in vegetables using the immunocompetition method. The results showed that the adsorption capacity of MIP to fenvalerate was 3.65, and the adsorption capacity on MIPFM (an electrospinning membrane containing the fenvalerate MIPs) was five times that of free MIP. The test strip showed good linearity with *R*^2^ = 0.9761 within the range of 50 μg/L-1,000 μg/L. In conclusion, substituting fenvalerate monoclonal antibodies with a molecularly imprinted electrospinning membrane is ideal for rapid onsite detection of pyrethroids.

## Introduction

Pyrethroid pesticides have the broadest spectrum of high efficiency, low residue, moderate toxicity, and are biodegradable in plants. It effectively prevents and controls the storage of insect pests and has become the preferred pesticide in today's agriculture (1, 2). Type II pyrethroid pesticides have good efficacy and stability and are widely used to protect crops against insects. The highest detection rate was reported in crops, followed by sediment, soil, and water. M-phenoxybenzoic acid (3 PBA) is a common metabolite of several pyrethroid insecticides (3). In addition, studies have shown that pyrethroids can trigger immune disorders, which may seriously impact the occurrence of immune-related diseases (4). Therefore, it is necessary to monitor pyrethroids in agri-products. Many countries and agencies have set tolerance or maximum residue limits (MRL) for pyrethroids based on the potential impact of a pesticide on humans, referred to as risk assessment. For instance, in the EU, the MRL of fenvalerate in vegetables ranged from 0.02 to 0.2 mg/kg. In the Codex Alimentarius (CAS), the MRL of fenvalerate in vegetables ranged from 0.05 to 3.0 mg/kg. In Japan, the MRL of fenvalerate in vegetables ranged from 0.05 to 3.0 mg/kg. In the Republic of Korea, the MRL in vegetables ranged from 0.05 to 10.0 mg/kg, whereas in China, the MRL in vegetables ranged from 0.05 to 10.0 mg/kg. As they are lipophilic, their absorption in the gastrointestinal and respiratory tracts is higher than on the skin. They are also transmitted directly and indirectly through the food chain and eventually threaten human health and life (5).

At present, pyrethroids are detected by various techniques, including chromatography (6–8), liquid chromatography-mass spectrometry (9–11), and immunological methods (12). Chromatography-mass spectrometry is mostly used as a confirmation method due to the separation power of chromatography combined with the high selectivity of mass spectrometry. It is a high-throughput hyphenated technique that can detect multiple analytes in a single run. However, the sample preparation is time-consuming, and the equipment is expensive and requires professional personnel to operate. Immunoassays (IAs) are bioanalytical techniques that measure enzymes and proteins quantitatively and qualitatively using a combination of antigens and antibodies. Conversely, the immunochromatographic assay is an analytical method that combines immunotechnology and chromatography using fluorescent substances or colloidal gold (13, 14) labels. It is widely used in various fields. However, pyrethroids are small molecules that do not trigger an immune response. Preparing antibodies in the mouse body and optimizing storage conditions increase the cost and necessitate the sacrifice of animals. Therefore, to meet the demand for large-scale assessments, it is necessary to establish a fast, sensitive, accurate, and low-cost onsite analysis to monitor food pyrethroids (15).

Molecularly imprinted polymers (MIPs) have an affinity for the predetermination and specific identification of a molecule in addition to their practicability. The polymer has a simple preparation process, low-cost, good mechanical and chemical stability, and long service life to satisfy the features of a specific recognition material with reasonable practicability (16–19). For instance, Azizollah Nezhadali et al. (17) prepared a selective molecularly imprinted polymer-based solid-phase extraction (MISPE) by bulk polymerization using fenvalerate as a template and polypyrrole (PPy) as a monomer. The detection and quantification limits of this method were 0.19 ng/g and 0.63 ng/g, respectively, with a relative standard deviation of 4.07%. The method is simple and utilizes the minimum solvent volume, replacing the traditional sample preparation. Furthermore, Wang et al. (20) established a highly selective and sensitive photochemical sensor, CdTe QDS-MIP, fixed on a paper-based working electrode to determine fenvalerate in fruits and vegetables. This method has higher photocurrent transfer efficiency and the advantages of simplicity, speed, and low cost compared with pure CdTe QDs. Moreover, Wang et al. (21) developed a method for the selective identification and detection of λ-cyhalothrin with 5-isothiocyanate (6) (FITC) and 3-aminopropyltriethoxysilane (APTS)/SiO2 combined with fluorescent MIP. This method eliminates the interfering substances in the sample and improves the detection limit. Due to its excellent properties, MIP has been used as a direct substitute for natural antibodies, receptors, and enzymes in submolecular biology (22). In this study, MIP was directly immobilized on a nitrocellulose (NC) membrane by streaking, and it flowed away with the solution. Therefore, a relatively fixed method is needed to firmly stabilize the MIP particles on the NC membrane. Electrospinning is a non-mechanical electrostatic technique that can spin nanofibers directly from the polymer solution (23). The obtained fiber has the advantages of a large specific surface area, high porosity, fast mass transfer rate, and easy separation (24–27). For instance, Xing et al. developed an atrazine (ATZ) molecularly imprinted nanofiber membrane based on electrospinning. The membrane has a large specific surface area, enhanced rebinding ability, and selective permeability of ATZ, as well as having strong practicability for separating ATZ in water samples (28). Furthermore, Ruggieri et al. synthesized MIPs using ATZ as a template and dispersed MIPs in an electrospinning solution dissolved in polystyrene to prepare a molecularly imprinted electrospun nanofiber membrane (MIM). The adsorption evaluation of ATZ, ATZ metabolites atalido-deisopropyl, carboxylin, linuron, atraton, and chlorpyrifos was carried out in distilled and river water. Compared with the adsorption capacity of MIP for these five pesticides, the adsorption capacity of MIM significantly increased (37). In addition, under low pesticide concentrations, the efficiency of commercial SPE was higher than that of the MIM prepared in this study; however, for complex matrices and high concentrations, MIM can replace commercial products well.

In our previous study, we developed a test strip based on surface molecularly imprinted electrospun affinity membranes to detect triazophos residues in water. We prepared the test line on a nitrocellulose membrane using a molecularly imprinted electrospinning technique. Then, we synthesized a triazophos hepten-murine IgG/fluorescein isothiocyanate conjugate (THBu-IgG-FITC) to directly compete with the target triazophos on the molecularly imprinted binding sites (29). However, the Stokes shift of the ordinary fluorescent group was 1–100 nm, the fluorescence lifetime was short, and quenching was easy, for example, for fluorescence quantum dots (30, 31). In comparison, time-resolved fluorescence immunoassays used trivalent rare-earth ions—such as Eu (III), Tb (III), and Sm (III)—as markers. These ions can produce high-intensity fluorescence and decay for a long time. Accordingly, short-delay measurement and interference with natural fluorescence can be eliminated, significantly improving the method's sensitivity (32, 33). It should be noted that the preparation of antibodies for pyrethroids is complicated; therefore, few strips can be used for rapid onsite detection. It is worth noting that there is no literature on the combination of molecularly imprinted electrospun nanofiber membranes and time-resolved fluorescence microspheres Eu. Hence, this study established a rapid determination method using a biomimetic immunoassay based on molecularly imprinted recognition material for the rapid and sensitive detection of fenvalerate in Brussels sprouts, cucumbers, and eggplants. Herein, time-resolved fluorescent latex microspheres Eu were labeled with a conjugate of fenvalerate hapten-IgG (FH-IgG). It competed with the pyrethroid molecule in the sample to bind specific binding sites on the MIPs. The method was evaluated from the quantitative standard curve, detection limit, linear range, and actual examples by exploring the assembly of the biomimetic immunofluorescence strip. The findings were compared with the results of GC–MS/MS.

## Experimental

### Materials

Ethyl chrysanthemate (95%) and dowtherm A were secured from Macklin Biochemical Co., Ltd. (Shanghai, China). 2,2'-Azobisisobutyronitrile (AIBN) was acquired from Bailingwei Co., Ltd. (Beijing, China). Acrylamide (AA), ethylene glycol dimethacrylate (EGDMA), cellulose acetate (CA), dicyclohexylcarbodiimide (DCC), EDC, N-hydroxysuccinimide (NHS), and BSA were acquired from Sigma–Aldrich, St. Louis, MO, USA. Fenvalerate was obtained from Dr. Ehrenstorfer (GmbH, Germany). Goat anti-mouse IgG and mouse IgG dry powder were purchased from Solarbio Technology Co., Ltd. (Beijing, China). Our laboratory provided fenvalerate haptens, whereas the NC membrane (NC95) was purchased from Sartorius Stedim in Germany. Glass fiber membrane RB45, absorbent paper SH27, and PVC base plate SMNF31-25 were obtained from Shanghai Jieyi Biotechnology Co., Ltd. (Shanghai, China). Sucrose, sodium chloride, sodium dihydrogen phosphate dihydrate, disodium hydrogen phosphate dodecahydrate, and Tween 20 were obtained from China National Medicines Corporation Ltd. (Beijing, China). Time-resolved fluorescent latex microspheres Eu (200 nm, wrapped by polystyrene) were obtained from Diibio Technology (Xiamen, China). Shanghai Yuanye Biotechnology Co., LTD supplied an MD3534-5 m dialysis bag. Deionized water was purified with a Milli-Q system (Millipore, Bedford, MA, USA). Acetone (purity ≥ 99.5%) was secured from Beijing Chemical Industry Group Co., Ltd., whereas HPLC-grade methanol (MeOH) was purchased from Fisher Scientific (Pittsburgh, PA, USA).

### Molecularly imprinted polymers

#### Preparation of dual-template imprinted polymers

**Figure 1:**
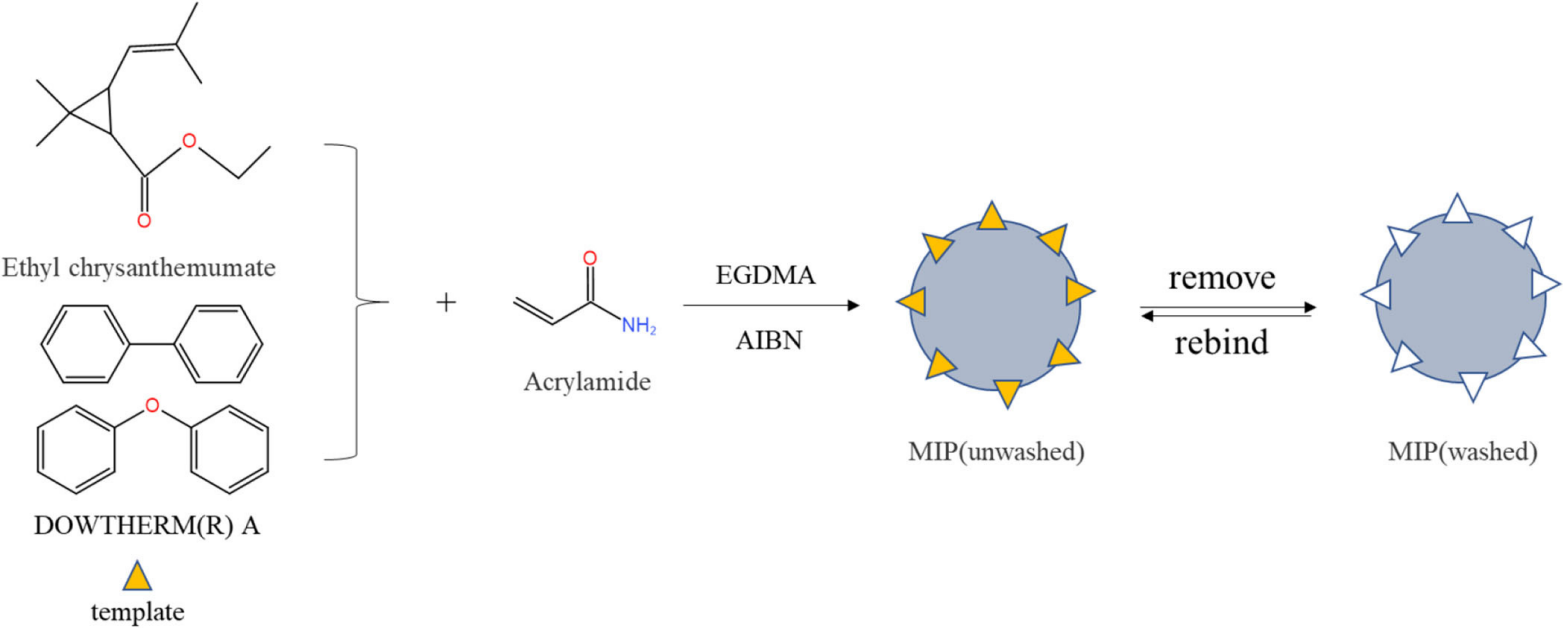
The synthesis process of MIP.

Molecularly imprinted polymers were prepared by precipitation polymerization (the brief process in Figure 1). Briefly, ethyl chrysanthemate (21.6 μL), 15.3 μL of dowtherm A and 43.236 mg of AA were dissolved in 50 mL of ACN in a round-bottomed flask and prepolymerized for 4 h in a water bath at room temperature. Then, 31.2 mg of AIBN and 282 μL of EGDMA were added separately to remove the oxygen from the solution by purging with nitrogen for 30 min and sealing. Afterward, the flask was placed in a water bath at 60 °C and shaken for 24 h at 150 rpm. After the polymerization reaction was completed, the mixture was centrifuged at 5,000 rpm for 5 min (Thermo Fisher Scientific, Waltham, MA, USA). Finally, the polymers were washed with methanol: acetic acid (*v/v*, 8:2) in a Soxhlet extractor until HPLC detected no template. The MIPs were washed several times with pure methanol, dried at 40°C, and stored at room temperature. Non-imprinted control polymers were the same as MIP conditions except without templates.

#### Binding experiments

According to the specific adsorption of MIPs, 20 mg of MIPs or NIPs and 1 mL of 40 mg/L fenvalerate were added to a centrifuge tube and oscillated for different times (0, 10, 20, 30, 60, 90, and 180 min) at room temperature. Next, the mixtures were centrifuged at 5,000 rpm for 3 min, and HPLC (Waters 2695 Alliance HPLC system with a 2489 UV detector, Waters Corporation, Milford, MA, USA) was used to determine the concentration at 235 nm for fenvalerate in the supernatant to assay the saturation adsorption time. Afterward, 20 mg of MIPs or NIPs was weighed in a 2 mL centrifuge tube, and 1 mL of fenvalerate at different concentrations (40, 60, 80, 100, 150, and 200 mg/L) was added and shaken well for 90 min. Finally, the mixtures were centrifuged at 5,000 rpm for 3 min, and HPLC was used to determine the concentration in the supernatant to calculate the saturated absorption amount. The adsorption capacity Q (mg/g) of MIP to fenvalerate was calculated using formula (1). The maximum apparent capacity and binding site were determined by the Scatchard equation (2).


(1) $$\begin{array}{r} Q=\left(C_{0}-C\right)V/m \end{array}$$

(2) $$\begin{array}{r} \frac{Q}{C}=\left(Q_{\max}-Q\right)/k_{d} \end{array}$$

where Q is the amount of MIP adsorbed on the target at equilibrium, C_0_ is the initial concentration of fenvalerate, C represents the final concentration in the supernatant at equilibrium, and V is the volume of fenvalerate (mL), with m referring to the amount of MIP added. Qmax is the maximum apparent adsorption capacity, and k_d_ is the equilibrium dissociation constant.

#### Specificity of PyMIP

The specific selectivity of PyMIP was evaluated using different concentrations of fenvalerate and the structural analogs silafluofen, sulfadiazine, ethiprole, and buprofezin at concentrations between 0.5 and 20 mg/L. Then, 5 mg of PyMIP and NIP were accurately weighed, and a 1 mL standard solution of fenvalerate (F), thiazone (B), acetonitrile (E), and sulfadiazine (Su) gradient series (0.5, 1, 2, 3, 5, 10, and 20 mg/L) was added. The solution was oscillated at room temperature for 1 h, centrifuged, and the supernatant was taken. After filtration through a 0.22 μm microporous membrane, the concentration of the target substance in the supernatant was determined by HPLC, the adsorption capacity was determined, and the specificity of the polymer was evaluated.

### Preparation of a molecularly imprinted test line on an NC membrane using electrospinning

#### Preparation of the electrospinning solution

A 15% electrospinning solution was prepared by adding 1.5 g of cellulose acetate (CA) to 12.7 mL of acetone and methanol (9:1, *v/v*). Then, the MIP dispersion solution and Tween-20 solution dissolved in methanol were added to the electrospinning solution. The solution was stirred at room temperature until the MIPs were evenly dispersed in the electrospinning solution (electrospinning solution: MIP dispersion solution: Tween-20, =1,000 μL:100 μL:10 μL, *v/v/v*) to obtain a uniform pyrethrin molecularly imprinted electrospinning solution.

#### Preparation of the T-line on the NC membrane by electrospinning

The laboratory electrospinning device was used to prepare nanofiber layers. The electrospinning solution was injected into a typical syringe, the 18-gauge needle was used as a nozzle, and the distance between the needle and the receiver was 16 cm. The spinning test was carried out under a voltage of 13 kV. At this point, the electrospinning membrane containing the fenvalerate MIPs (MIESM) was formed on foil paper and then placed in an oven at 37°C for drying. After removing the paper, the film was cut into a shape similar to the test line (T-line) in the lateral flow test strips and stuck on scotch tape for later use.

### Preparation of the immunochromatographic test strip

#### Synthesis of the FH-IgG conjugate

The synthetic method was moderately modified based upon previous work carried out by Yahui He as follows: 10 mg of fenvalerate hapten (FH), 4.6 mg of N-hydroxysuccinimide (NHS), and 8.24 mg of dicyclohexylcarbodiimide (DCC) were dissolved in a 5 mL glass bottle with 0.5 mL of anhydrous DMF. The reaction mixture was stirred overnight at room temperature and then centrifuged at 4,000 rpm for 1 min. The supernatant was collected and slowly added to 1.5 mL of PBS (0.01 mol/L) containing 4 mg of mouse IgG and stirred at room temperature for 3 h. All of the above solutions were transferred to a dialysis bag. Finally, the solution was dialyzed several times against 0.01 mol/L PBS solution (pH = 7.4) at 4 °C until the dialysate was clear. Afterward, the conjugate (FH-IgG) was freeze-dried in a freeze-dryer for 24 h, stored at −20 °C, and then set aside.

#### Time-resolved fluorescent microsphere-FH-IgG conjugates

A 100 μL time-resolved fluorescence microsphere Eu was added to 900 μL of labeled buffer (50 mM 2-morpholinoethanesulfonic acid (MES), pH = 6.0). To activate the carboxyl groups on the surface of time-resolved fluorescence microspheres Eu, 10 μL and 5 μL of 20 mg/mL NHS and EDC were added sequentially and mixed at room temperature for 20 min. After the solution was centrifuged at 9,000 rpm for 15 min and cleaned by MES several times, 0.5 mg of FH-IgG was added and kept at room temperature for 2 h. Afterward, 100 μL of 20 mg/mL BSA (dissolved in 0.05 M PBS buffer solution) was added to the solution and mixed at room temperature for 1 h. The obtained solution was centrifuged at 9,000 rpm for 15 min. The fluorescent microspheres were precipitated twice to remove the unbound antibodies. The fenvalerate hapten-IgG-fluorescent microsphere labeling complex (FH-IgG-Eu) was obtained and stored at 4°C for further experimental work.

#### Preparation and assembly of the immunochromatographic strip assay

##### Assemble biomimetic immunochromatographic strip test

**Figure 2:**
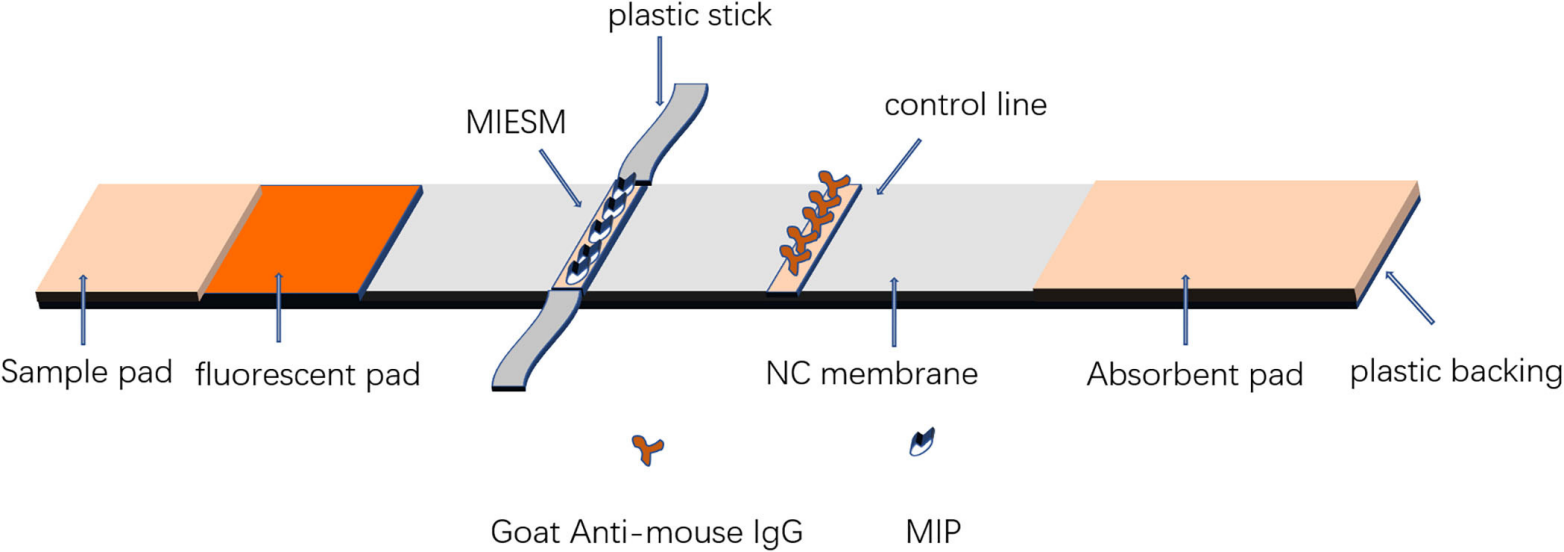
Assembly of the biomimetic immunochromatographic strip assay.

The composition of the test strip is shown in Figure 2. The fluorescence was sprayed on the mat at a speed of 0.5 μL/mm by the injector and then dried in the oven at 37°C for further use. Goat-mouse IgG was coated on a nitrocellulose (NC) membrane as a control (C) line by an HM3030 XYZ 3D membrane spraying instrument (Shanghai Jieyi Biotechnology Co., Ltd., Shanghai, China) at a proper jetting rate. The coated membrane and sample pad treated with blocking buffer were dried overnight at 37°C. Then, the NC membrane, sample pad, and absorbent were assembled on a fluorescent plastic backing and cut to 4 mm in length. Afterward, the electrostatic fiber membrane was removed from the foil paper and stuck on the sticker, and then the area of the MIP membrane was cut into pieces 2 mm in width, 4 mm in length, and then stuck 5 mm from the C-line as the T-line.

##### Detection principle of the immunochromatographic strip test

**Figure 3:**
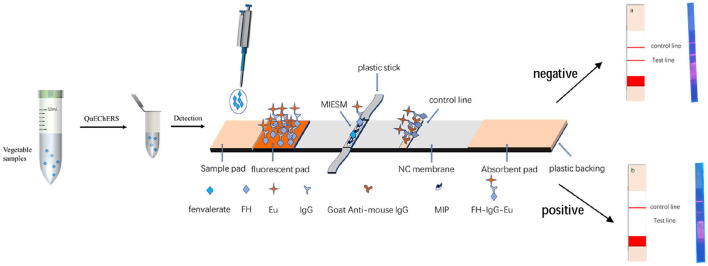
Detection principle.

The MIP was fixed on the NC membrane by electrospinning (alternative to antibody) as the T-line and goat-mouse IgG as the C-line immobilized by the instrument and sprayed the FH-IgG-Eu solution onto a mat as a fluorescent mat. As shown in Figure 3, when fenvalerate was dripped onto the sample pad, it moved to the fluorescent mat, and the FH-IgG-Eu was dissolved in the buffer solution and flowed to the NC membrane together. The target fenvalerate and the FH in FH-IgG-Eu could compete for the binding site at the MIPs so that the fluorescence intensity of the T-line forms an inverse relationship with the concentration of fenvalerate. The remaining fluorescence probes are transported to the C-line and combined with secondary antibodies to effectively control the quality of the label flow strips. Finally, the immunofluorescence card reader (Beijing Qinbang Biology Co., LTD. Beijing, China) read T, C, and T/C values according to the intensity of T-line fluorescence and T/C value to be quantified.

#### Immunochromatographic strip optimization procedure

##### The fixation method of the T-line and the use of fluorescent microspheres

Because MIPFM replaces the T-line, its fixation needs to be optimized to do the T-line work normally and reduce agglomeration. Therefore, the following two methods were tested. One method involved not cutting the NC membrane directly to glue the MIPFM and fixing it on the NC membrane as a T-line. Another way required cutting the NC membrane and sticking the MIPFM with foil paper as a T-line.

Two methods were tested: one mixed the fluorescent probe and buffer solution and dropped it into the test (way 1), and the other used the fluorescent pad for the test (way 2). According to the calculation, the spraying amount of the fluorescent pad was approximately 2 μL/4 mm. Thus, 2 μL of fluorescent probes were added to 100 μL of buffer solution in the direct drip method.

##### Optimization of the amount of MIP, FH-IgG, and fluorescence

Taking the FH-IgG labeling amount, fluorescent pad spraying amount, and MIP content in the T-line as three factors, we designed the test to fulfill the optimal test parameters of the strip. The secondary antibody concentration in the C-line was fixed at 2 mg/mL. As described in Section GC–MS/MS, the fluorescence immunocard reader was used to read the T/C values under the negative condition. At the same time, 100 μL of fenvalerate standard solution (500 μg/L) was dropped to compete with MIP binding sites as a positive control. Formula (3) was used to calculate the competitive inhibition rate. The optimum experimental conditions were selected as the appropriate T-line fluorescence intensity and the maximum rate of competitive inhibition. Each experiment was reproduced five times, and the final results were expressed as an arithmetic mean.


(3) $$\begin{array}{r} {\left(1-\frac{B}{B_{0}}\right)}^{*}100\% \end{array}$$

where B is the T/C value under positive conditions and B_0_ is the T/C value under negative conditions.

##### Optimization of the concentration of secondary antibodies

Sheep anti-mouse IgG at concentrations of 1, 2, 3, and 4 mg/mL was fixed on the NC membrane to assemble the strips. The optimal concentration of secondary antibodies was selected according to the competitive inhibition rate and the brightness of the lines on the strips.

##### Optimization of the buffer solution

The buffer solution was vital for the fluorescence microspheres on the NC membrane. The buffer solution affects whether fluorescence microspheres climb. In this experiment, eleven different buffer solutions were prepared to select the appropriate solution (Supplementary Table S1).

##### Optimization of the organic solvent tolerance of the immunochromatographic strip test

Because fenvalerate is insoluble in water, organic solvents were used for reconstitution. Herein, we tested acetonitrile, methanol, isopropanol, ethanol, ethyl acetate, acetone, and *n*-hexane. Furthermore, acetone and *n*-hexane can dissolve fenvalerate directly through GC/MS. Due to the limited IgG and NC membrane tolerance to organic solvents, it is necessary to optimize the addition amount. Thus, we set the amounts to 1, 5, 10, 15, and 20% for optimization.

##### Performance of the method

To estimate the performance of the strip, the buffer solution was used to dilute the fenvalerate standard (dissolved in acetonitrile) to concentrations of 0, 20, 50, 100, 200, 500, 800, and 1,000 μg/L, and 100 μL was added to the assembled strip. After 15 min of reaction in a dark environment, the values were read, and each concentration was repeated five times. The Log10 value of the concentration of fenvalerate standard solution was used as the X-axis, and the corresponding T/C value was used as the Y-axis. The T/C value decreased gradually with increasing concentrations of the standard solution. The IC50 and the limit of detection (LOD), defined as the fenvalerate concentration with 10% inhibition, were computed by the standard curve and used as reference standards of the sensitivity.

### Sample preparation

Vegetables, including Brussels sprouts, cucumbers, and eggplants, were procured from local supermarkets in Beijing. The homogenates were pretreated with QuEChERS. A10 samples (accurate to 0.01 g) were weighed into a 50 mL centrifuge tube, and the fenvalerate standard was spiked at 100, 500, and 1,000 μg/L. After withstanding for 30 min, 10 mL of acetonitrile, 4 g of anhydrous magnesium sulfate, 1 g sodium chloride, 1 g sodium citrate, and 0.5 g disodium hydrogen citrate were added, shaken vigorously for 1 min, and centrifuged at 4,200 r/min for 5 min. A 3 mL supernatant was added to 450 mg of anhydrous magnesium sulfate and 150 mg of PSA, followed by vortex mixing for 1 min and centrifugation at 4,200 r/min for 5 min. The supernatant was filtered through microporous filtration membranes (0.22 μm) for analysis.

#### Test strip

First, 1 mL of supernatants were dried with nitrogen in a centrifuge tube, then 50 μL acetonitrile was added into that centrifuge tube for redissolution, after which a 1,950 μL buffer solution was also added into that centrifuge tube to diluent the solution for the strip determination. Measure 100 μL of the solution and drop it onto the strip for the test.

#### GC–MS/MS

0.5 mL of supernatants were dried with nitrogen in sample vials, and then 1 mL of *n*-hexane was added to the sample vial to redissolve fenvalerate for detection by GC–MS/MS (Shimadzu GCMS-TQ8040 gas mass spectrometry tandem mass spectrometry, Shimadzu, Japan). The capillary column was a DB-5MS chromatographic column (5% phenyl and 95% polysiloxane stationary phase, 30 m × 0.25 mm × 0.25 μm). The GC oven temperature program was as follows: 50°C was raised to 150°C at a rate of 25°C/min and then raised to 300°C at a rate of 35°C/min and held for 4 min.

## Results and discussion

### Characterization of MIPs and NIPs

#### Morphology of PyMIP and NIP

**Figure 4:**
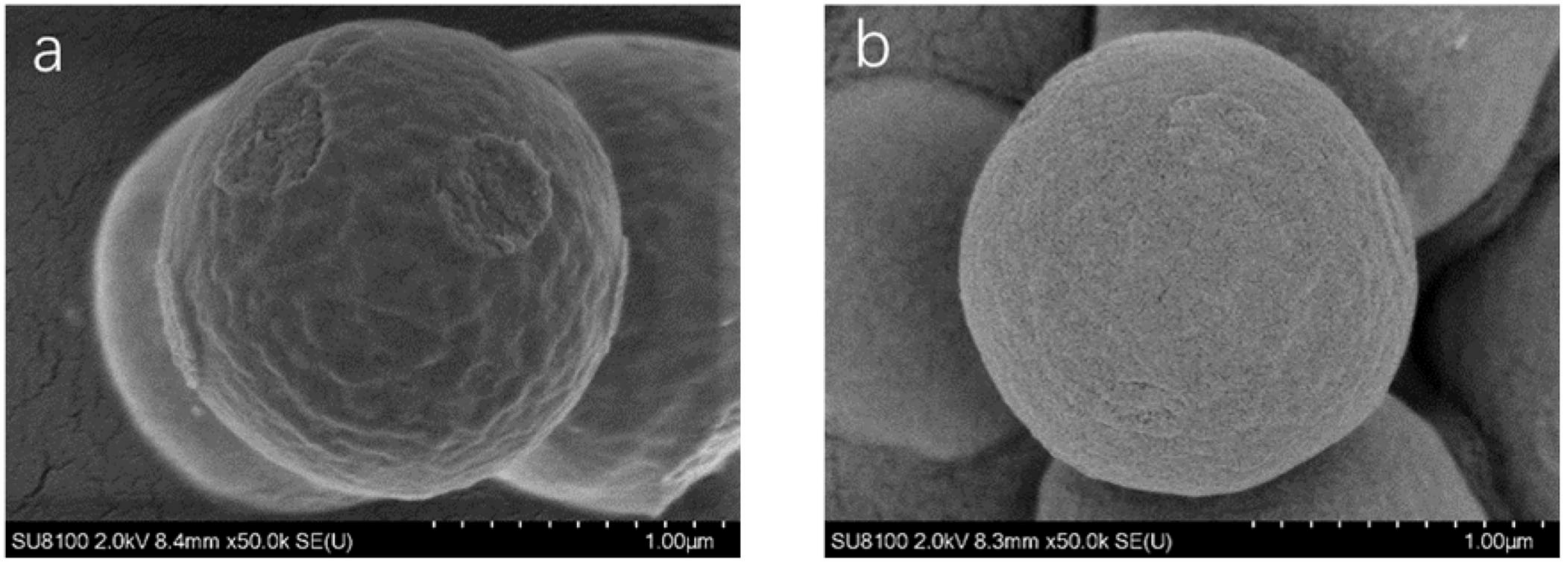
Scanning electron micrographs of MIP **(a)** and NIP **(b)**.

**Figure 5:**
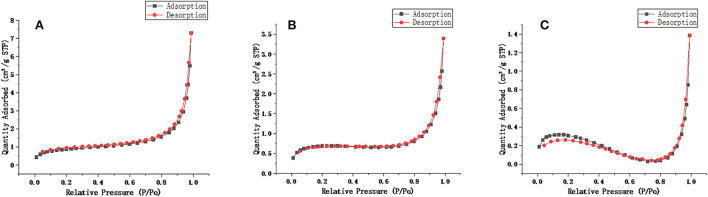
Linear isotherm plot of **(A)** washed MIP. **(B)** Unwashed MIP. **(C)** NIP.

Scanning electron microscopy (SEM, SU8020, Hitachi, Japan) was used to characterize the synthesized molecularly imprinted polymers. As shown in Figure 4, the NIP was similar to the MIP, but the spheres were smoother, and the particle diameters of the MIP and NIP were approximately 1.3 μm. The surface area measurements of unwashed, washed MIP and NIP were performed and were 2.1170, 2.9272, and 0.8669 m^2^/g, respectively. The BET areas of MIPs are 3.37 and 1.3 times higher than those of NIPs and MIPs without elution, respectively. From Figure 5, the pore volumes of MIPs are 6.1 and 2.3 times higher than those of NIPs and MIPs without elution, respectively. These results indicate that after eluting the templates, many mesopores of the templates are imprinted on MIPs. MIPs with more imprinted cavities and active sites would exhibit greater adsorption capacity and a greater number of combined target molecules than NIPs and MIPs without elution.

**Figure 6:**
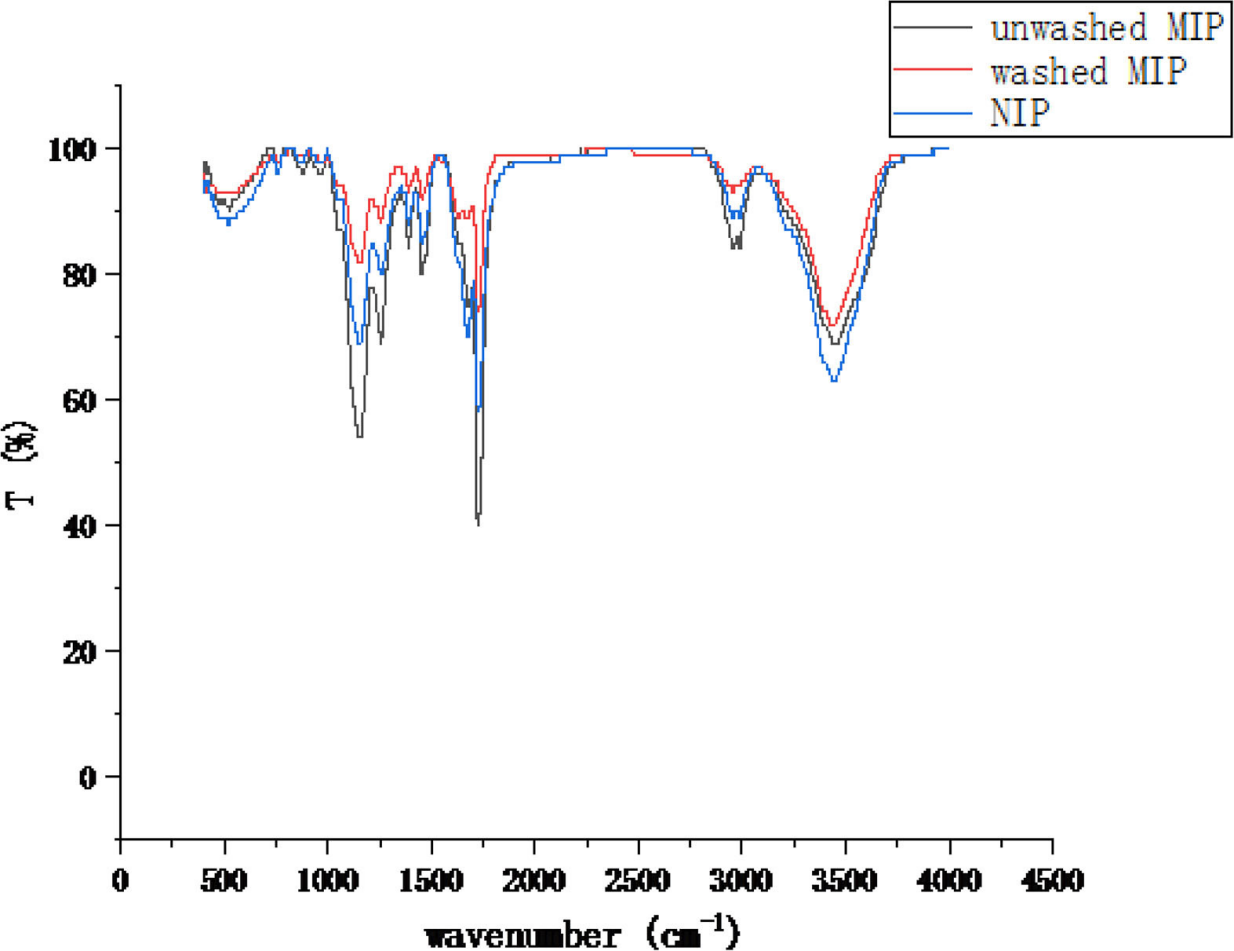
FTIR of unwashed MIP, washed NIP, and NIP.

To further confirm the chemical bonds and functional groups of the MIP, the materials were characterized by FTIR spectrometry (Figure 6). The characteristic peaks of the unwashed MIP at wavelengths of 1,160 cm^−1^ and 1,260 cm^−1^ are higher than those of NIP and washed MIP, which might be attributed to the C-H in-plane bending vibration of the ester group of ethyl chrysanthemate and dowtherm A, respectively. Strong characteristic peaks at 1,730 cm^−1^ and 3,450 cm^−1^ are assigned to the stretching vibration of amide C=O and O-H or N-H of acrylamide, respectively. In sum, MIPs were synthesized successfully.

#### Adsorption and specific selectivity analysis of PyMIP

**Figure 7:**
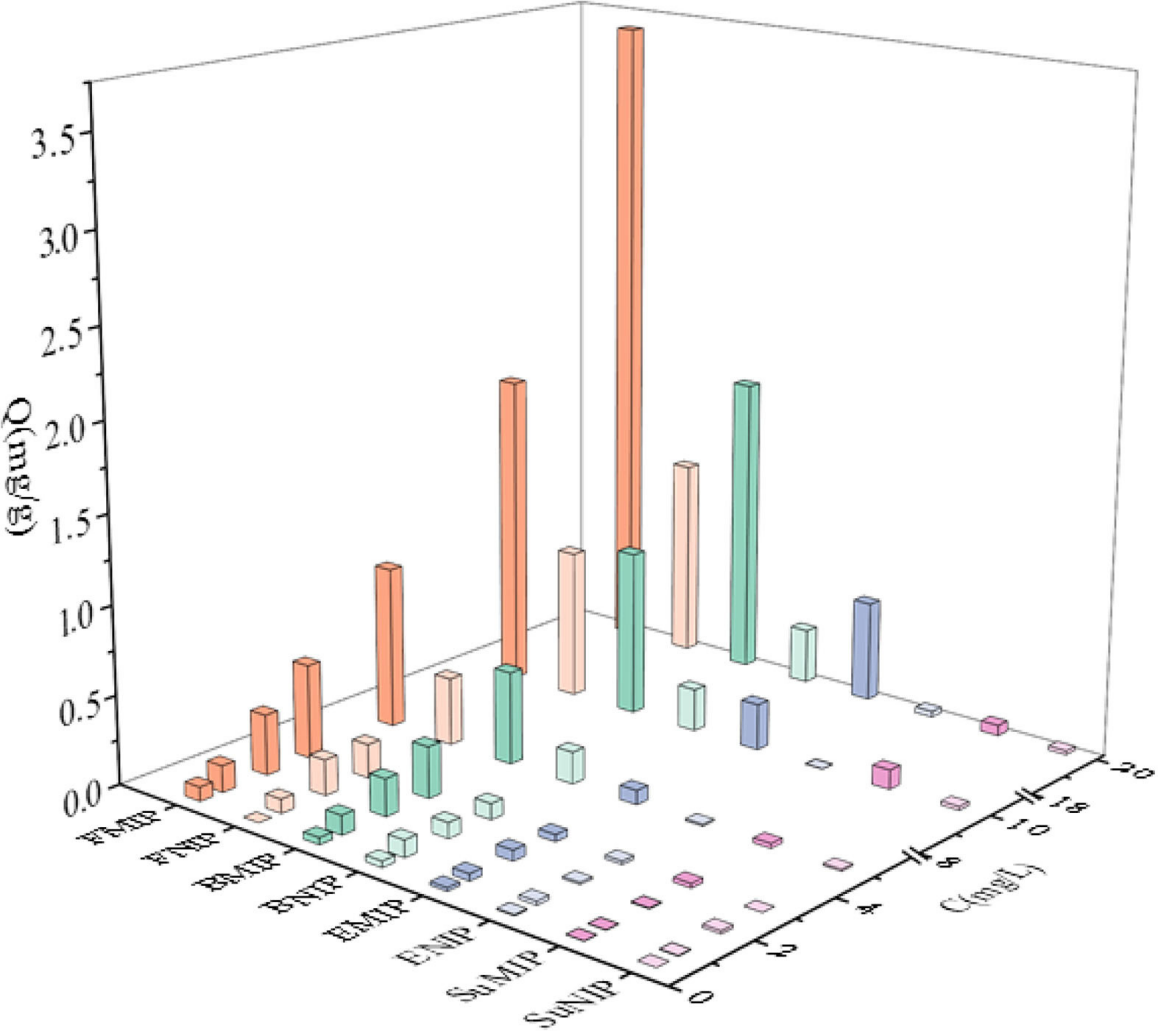
Special adsorption of PyMIP and NIP. FMIP, fenvalerate; MIP, BMIP: buprofezin; MIP, EMIP, ethiprole; MIP, and SuMIP, sulfadiazine MIP.

Through the dynamic and isothermal adsorption experiments of fenvalerate by MIP and NIP. Supplementary Figure S1A shows that the adsorption capacity of MIP and NIP concerning fenvalerate increases gradually with increasing time, and the saturation adsorption time was determined to be 90 min. Supplementary Figure S1B shows that the adsorption capacity of MIP and NIP increased with increasing fenvalerate concentration. MIP had a higher adsorption capacity for fenvalerate than NIP. Therefore, MIP has specific adsorption to fenvalerate. As shown in Supplementary Figure S1C, Q/C and Q were linearly related, suggesting that MIP had a single binding site per template molecule. According to formulas (1) and (2), Qmax is 3.65 mg/g, and Kd is 47.62 mg/L, which are higher than those in the literature (34). By comparing the experimental results with those in other literature (Table 1), we concluded that the MIPs have a high adsorption capacity. The PyMIP exhibited specific adsorption of fenvalerate better than the other pesticides and had apparent selectivity for fenvalerate (Figure 7).

**Table 1:** Comparison of MIPs with other literature.

**Imprinting system**	**Synthetic method**	**Imprinting factor**	**Qmax μg/g**	**References**
Ethyl chrysanthemate and dowtherma: MAA	precipitation polymerization	6.12	3650.0	This paper
Fenvalerate: Pyrrole	bulk polymerization	1.09	1084.4	(17)
Fenvalerate: MICOFs	Schiff reaction	4.60	9657.7	(35)
Fe_3_O_4_-NH_2_@GO@MIP: 3-PBA	surface polymerization	1.78	91700.0	(36)
Phenyl ether-biphenyl eutectic: MAA	atom transfer radical polymer ization	1.92	2300.0	(34)

### Characterization of MIP electrospun membranes

The electrospun membrane was obtained under 15% electrospinning solution mass concentration, an 18 G spinning needle, a 16 cm distance from the receiver, and 13 kV voltage. As shown in Supplementary Figure S2, the fiber diameter of the spinning membrane (MIPFM) under this condition is uniform.

Compared to the adsorption properties of MIPFM and MIP, the adsorption capacity of fenvalerate on MIPFM was five times that of free MIP, as shown in Supplementary Figure S3. This may be because MIPs wrapped in electrospinning solution are more dispersed, which fully exposes the adsorption site and improves the adsorption capacity of fenvalerate.

### High-resolution mass spectral characterization of FH-IgG-Eu

By comparing the *m/z* values of the mouse antibody, fenvalerate hapten, and FH-IgG, the success of FH-IgG conjugation was confirmed, and the conjugation ratio was estimated. As shown in Supplementary Figure S4, the *m/z* value of antibody (b) was 71232.385, and the *m/z* value of the coupling between the two (c) was 81044.724. In addition, the molecular mass of fenvalerate hapten (a) was 499, and the *m/z* value of the connection between the two was higher than that of the antibody. Therefore, it was suggested that the antibody and fenvalerate hapten had linked successfully, and the connection ratio was 1:20.

### Optimization of immunochromatographic strip parameters

#### The fixation of the T-line and the usage of fluorescent microspheres

In method 1, the MIPFM was stuck at the cutting point when the NC membrane was cut. The cutting point has much fluorescence, making the fluorescence at the C-line fragile and in the T-line very strong but prone to false-negative results. However, method 2 could avoid the phenomenon of fluorescence agglomeration at the T-line. Thus, method 2 was selected for the subsequent experiments. The results showed that way 1 had a high background value when we dropped the fluorescent probe directly. However, the opposite result was observed in way 2, which used a fluorescence pad. When the buffer solution had been chromatographed, the background was spotless; therefore, we selected the second method to use a fluorescence pad for further experiments.

#### Optimization of the amount of MIP, FH-IgG, and fluorescence

The results showed that the fluorescence intensity of the T-line was strong. The inhibition rate was 78.2% when the FH-IgG labeling amount was 0.5 mg, the spraying amount of the fluorescence pad was 5 μL/cm, and the MIP amount of the T-line was 10 mg/mL. We also found that the FH-IgG labeling amount was 0.5 mg, with the spray amount of the fluorescent pad being more than 5 μL/cm. The T-line value was higher when the spraying amount was 5 μL/cm. However, due to the high cost of FH-IgG, a spraying amount of fluorescence of 5 μL/cm was selected as the optimal experimental condition.

#### Optimization of the concentration of secondary antibodies

Under ultraviolet light, the color of the line is light or dark and can be misread. As shown in Supplementary Figure S5, when the secondary antibody concentration was 2 mg/mL, the competitive inhibition rate was 79.4%. The C-line had bright red fluorescence under the UV lamp. On the other hand, when the secondary antibody concentration was 1 mg/mL, the color of the C-line was relatively lighter and difficult to distinguish. Furthermore, when it was 3 and 4 mg/mL, the competitive inhibition rate was high; however, the color of the C-line was darker and considerably wider. The parallelism of reading values by card readers was substantially different. Therefore, 2 mg/mL was chosen as the optimal concentration of the C-line.

#### Optimization of the buffer solution

In this experiment, eleven different buffer solutions were prepared to select the most suitable one. When 0.02 molar (M) phosphate buffer (PB)+20% Tween-20 was used as a buffer solution, the brightness of the T- and C-lines was the best, and the edges of the T- and C-lines were smooth under UV; hence, it was used as a buffer solution. However, pyrethroids have low water solubility and are hydrophobic. We needed an organic reagent that could dissolve in water, so the influence of the organic reagent on the strip was determined when 5% acetonitrile was added to the buffer solution. Without adding Tween-20, we found that the brightness of the T- and C-lines was relatively shallow compared to that of Tween-20, even though there was no brightness on the T- and C-lines in many repeated experiments. When a certain amount of sucrose was added to the buffer solution, the brightness of the T- and C-lines was not changed significantly. Thus, the method without sucrose was chosen to simplify the experimental process. It must be noted that the addition of cations would have a particular influence on the charge balance of time-resolved fluorescence microspheres, so PB buffer was selected as the buffer solution. In conclusion, 0.02 m PB+20% Tween-20 was taken as the optimal buffer solution, and acetonitrile, the tolerable amount of organic solvent in the strip, was added to determine the analytes in the matrices. Then, the strip was measured by diluting the analytes with buffer solution.

#### Optimization of the organic solvent tolerance of the immunochromatographic strip test

The results showed that the darker the background color is, the more significant the impact on T and C read values when the competitive inhibition rate is higher than 60%. False-positive or false-negative results were judged when the background color was darker than the T-line. Therefore, acetonitrile was selected for further testing.

By comparing the amount of acetonitrile added in different ratios under a UV lamp, the brightness of the T and C lines gradually decreases with increasing acetonitrile content. The background of the strip was red, and the value read by the card reader of the strip was no longer accurate, resulting in incorrect interpretation. When the amount of acetonitrile was more than 5%, the edge of the T-line and C-line strips became no longer apparent. Hence, the highest organic solvent tolerance of the strip was 5%.

#### Method validation

**Figure 8:**
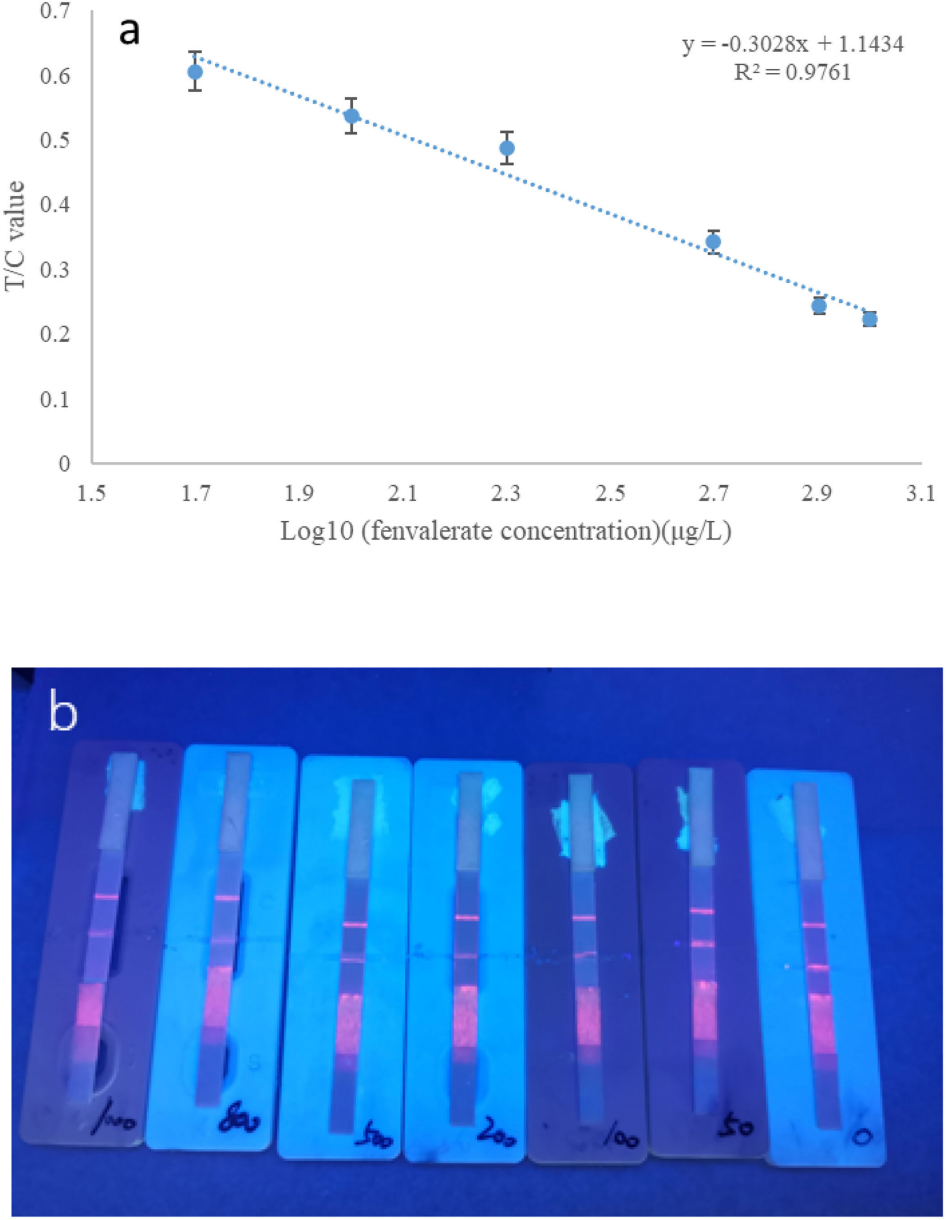
**(a)** Standard curve of fenvalerate measured by strip. **(b)** Different concentrations of fenvalerate (from right to left 0, 50, 100, 200, 500, 800, and 1,000 μg/L).

Under the optimal conditions stated above, a time-resolved fluorescent strip was used to detect different concentrations of fenvalerate standard. The buffer solution was used to dilute a series of standard concentrations of 50, 100, 200, 500, 800, and 1,000 μg/L. The results are shown in Figure 8. The Log10 value of the concentration of fenvalerate standard solution was used as the X-axis, and the corresponding T/C value was used as the Y-axis. The T/C value decreased gradually with increasing concentrations of the standard solution. The two are linear (y = −0.3028x+1.1434) with a coefficient of determination of *R*^2^ = 0.9761 (Figure 8a). When the concentration of the standard solution was 50 μg/L (Figure 8b), the competitive inhibition rate was 61.4%, a value higher than 60%. Hence, 50 μg/L was considered the detection limit for fenvalerate, which was lower than the MRLs of fenvalerate in Brussels sprouts, cucumbers, and eggplants in the Chinese standard (GB2763-2021).

#### Analysis of fenvalerate in real samples

The developed method was evaluated by analyzing fenvalerate in Brussels sprouts, cucumbers, and eggplants. Different concentrations of fenvalerate at 100 μg/L, 500 μg/L, and 1,000 μg/L were added to the blank matrices, and sample pretreatment was carried out as described in Section *Sample preparation*. Each concentration level was tested at least three times using a strip of test paper. The recovery rates ranged from 68.7to 109.2%, with RSDs between 1.8 and 14.4%. This finding implies that the strip could detect fenvalerate. To further verify the applicability of this method, ten cabbage samples were tested, and none of the samples tested positive for fenvalerate residues. This finding was similar to the results of GC–MS/MS.

## Conclusions

A lateral flow test strip was successfully designed based on molecularly imprinted and electrospun techniques to rapidly detect fenvalerate residues in vegetables. Unlike the traditional test strip for pesticide detection, our innovation lies in a molecularly imprinted electrostatic spinning film instead of a monoclonal antibody. The results were calculated using T/C values and target concentration curves using a fenvalerate hepten-IgG-Eu fluorescent probe competing with fenvalerate in the sample for specific recognition sites on MIPs. The method showed a good linear relationship in the 50–1,000 μg/mL concentration range, with a detection limit of 51.29 μg/mL. This is an attempt to reduce animal sacrifice and experimental costs, and the method is relatively simple and fast and can be used for rapid detection in the field.

## Data availability statement

The original contributions presented in the study are included in the article/Supplementary material, further inquiries can be directed to the corresponding author/s.

## Author contributions

LZ: data curation and writing-original draft. YZ: data curation and resources. HS: funding acquisition and writing-review and editing. MJ and FJ: supervision and validation. MX and JS: review. JW: project administration. AMAE-A: writing-review and editing. YS: formal analysis, methodology, and writing-review and editing. All authors contributed to the article and approved the submitted version.

## Conflict of interest

The authors declare that the research was conducted in the absence of any commercial or financial relationships that could be construed as a potential conflict of interest.

## Publisher's note

All claims expressed in this article are solely those of the authors and do not necessarily represent those of their affiliated organizations, or those of the publisher, the editors and the reviewers. Any product that may be evaluated in this article, or claim that may be made by its manufacturer, is not guaranteed or endorsed by the publisher.
