# Supplementary material for: Development of a time-resolved fluorescence microsphere Eu lateral flow test strip based on a molecularly imprinted electrospun nanofiber membrane for determination of fenvalerate in vegetables
Source: Front Nutr. 2022 Sep 20;9:957745. doi: 10.3389/fnut.2022.957745 (PMC9531596; doi:10.3389/fnut.2022.957745)
Supplement: Supplementary file 1 [file Presentation_1.pdf]

Figures List:

Fig.S1. Dynamic and static adsorption of fenvalerate on MIP(a), NIP(b), and Scatchard plot for MIP(c)

Fig.S2. SEM images of electrospun membrane

Fig.S3. Adsorption capacity of MIPFM and MIP

Fig.S4. High resolution mass spectrometry of fenvalerate hapten (a), antibodies (b), and FH-IgG(c)

Fig.S5. Positive C value and competitive inhibition rate under different concentrations of secondary antibodies

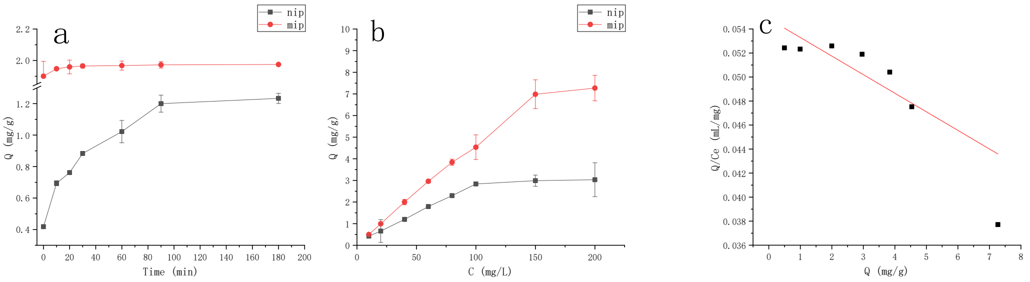

Fig. S1. Dynamic(a) and static adsorption(b) of fenvalerate of MIP and NIP, (c)scatchard plot for MIP

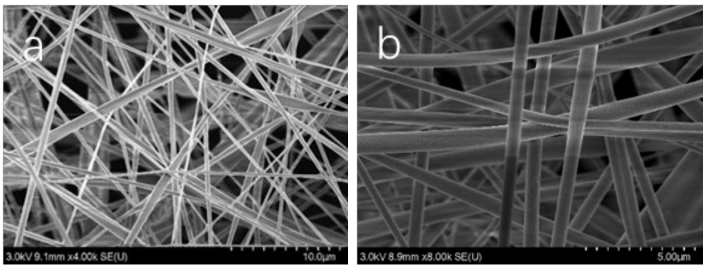

Fig.S2 SEM images of electrospinning membrane

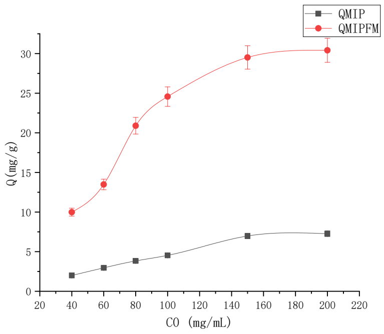

Fig.S3 the adsorption capacity of MIPFM and MIP

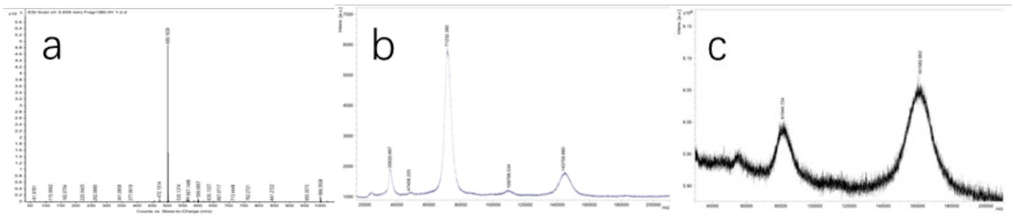

Fig.S4 High resolution mass spectrometry of fenvalerate hapten(a), antibodies(b) and FH-IgG(c)

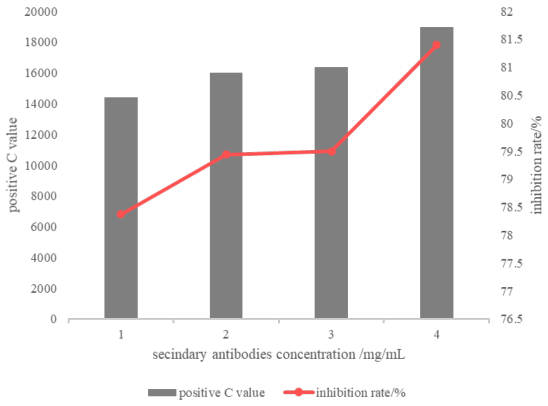

Fig.S5 Positive C value and competitive inhibition rate under different concentrations of secondary antibodies
